# Make a higher impact: Publish in Heliyon Nursing and health professions

**DOI:** 10.1016/j.heliyon.2024.e31295

**Published:** 2024-05-16

**Authors:** Carolyn Mackintosh Franklin

**Affiliations:** University of Manchester, UK

I am pleased to announce the latest exciting development from Cell Press and *Heliyon*, introducing **Heliyon Nursing and Health Professions** as the latest section of *Heliyon* specifically dedicated to publishing output of scientific value and relevance to nursing and a broad range of healthcare professionals. This creates an additional invaluable opportunity for all nursing and healthcare professionals to see their work published in a high impact, internationally recognised quality, open access journal.

As Section Editor of Heliyon Nursing and Health Professions, my aim is to facilitate the timely publication of papers on a broad range of nursing and health professions related subject areas. Additionally it will offer authors from many countries and with highly varied interests a new high quality open access home. While readers will also have access to a broad range of studies with direct relevance to education, management, research and clinical practice.

In this era of abundant digital information, the need to provide high quality evidence-based knowledge which is universally accessible is paramount. This knowledge can then in turn be used proactively in the promotion of best practice. Publication in **Heliyon Nursing and Health Professions** provides both authors and readers with high levels of confidence in the quality and accuracy of scientific papers and ethical rigour in research processes.

Publishing in **Heliyon Nursing and Health Professions** is a great opportunity for authors and researchers both young and experienced to see their work published, read and potentially utilised towards the ongoing enhancement of practice, supported by our constantly expanding team of expert Associate Editors.

I

The **Heliyon Nursing and Health Professions** section welcomes contributions across, but is not limited to, the following professional fields and research topics:

Nurses, Physiotherapists Pharmacists, Occupational Therapists, Physicians, Healthcare assistants, Medical education, Physician associates and diverse health researchers.

Research topics are broad ranging across the fields of clinical practice, healthcare management, research methodologies, and education.

## CRediT authorship contribution statement

**Carolyn Mackintosh Franklin:** Writing – review & editing.

## Declaration of competing interest

The authors declare that they have no known competing financial interests or personal relationships that could have appeared to influence the work reported in this paper.

